# Multi-round recycling of green waste for the production of iron nanoparticles: synthesis, characterization, and prospects in remediation

**DOI:** 10.1186/s11671-023-03784-x

**Published:** 2023-02-09

**Authors:** Andrea Rónavári, Margit Balázs, Árpád Szilágyi, Csaba Molnár, Márta Kotormán, István Ilisz, Mónika Kiricsi, Zoltán Kónya

**Affiliations:** 1 Department of Applied and Environmental Chemistry, University of Szeged, Szeged, Hungary; 2 Division for Biotechnology, Bay Zoltan Nonprofit Ltd. for Applied Research, Szeged, Hungary; 3 Department of Biochemistry and Molecular Biology, University of Szeged, Közép fasor 52, Szeged, 6726 Hungary; 4 Koch Institute for Integrative Cancer Research, Massachusetts Institute of Technology, Cambridge, MA USA; 5 Institute of Pharmaceutical Analysis, University of Szeged, Szeged, Hungary; 6 ELKH-SZTE Reaction Kinetics and Surface Chemistry Research Group, Szeged, Hungary

**Keywords:** Iron nanoparticles, Green synthesis, Multi-round waste recycling, Sustainable nanotechnology, Renewable raw material

## Abstract

Due to the widespread applications of metal nanoparticles (NPs), green synthesis strategies have recently advanced, e.g., methods that utilize extracts made from different plant wastes. A particularly innovative approach to reducing large amounts of available household/agricultural green wastes is their application in nanoparticle generation. Regarding this, the aim of our work was to examine the possibility of upgrading green nanoparticle syntheses from an innovative economic and environmental point of view, namely by investigating the multiple recyclabilities of green tea (GT), coffee arabica (CA), and Virginia creeper (*Parthenocissus quinquefolia*) (VC) waste residues for iron nanoparticle (FeNPs) synthesis. The plant extracts obtained by each extraction round were analyzed individually to determine the amount of main components anticipated to be involved in NPs synthesis. The synthesized FeNPs were characterized by X-ray powder diffraction and transmission electron microscopy. The activity of the generated FeNPs in degrading chlorinated volatile organic compounds (VOC) and thus their future applicability for remediation purposes were also assessed. We have found that VC and especially GT residues could be reutilized in multiple extraction rounds; however, only the first extract of CA was suitable for FeNPs’ generation. All of the obtained FeNPs could degrade VOC with efficiencies GT1-Fe 91.0%, GT2-Fe 83.2%, GT3-Fe 68.5%; CA1-Fe 76.2%; VC1-Fe 88.2%, VC2-Fe 79.7%, respectively, where the number (as in GT3) marked the extraction round. These results indicate that the adequately selected green waste material can be reutilized in multiple rounds for nanoparticle synthesis, thus offering a clean, sustainable, straightforward alternative to chemical methods.

**Graphical abstract:**

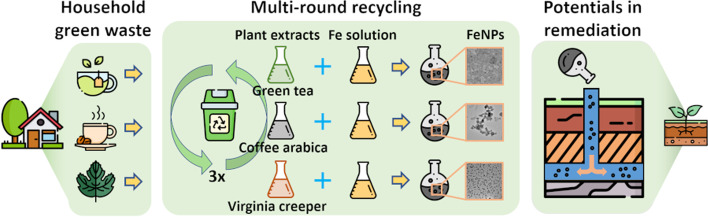

**Supplementary Information:**

The online version contains supplementary material available at 10.1186/s11671-023-03784-x.

## Introduction

Urbanization and improper industrialization combined with excessive waste disposal ultimately led to continuous contamination of groundwater and soil, which has become a serious issue and concern worldwide; therefore, the development of efficient novel remediation approaches is compulsory [1]. In the last decade, various types of nanoparticles (NPs)—such as carbon-based-, ceramic-, semiconductor-, polymeric-, lipid-, and metal NPs [2]—have gained attention and were implemented for various purposes. Among these, metal NPs such as FeNPs, AgNPs, and TiO_2_NPs were extensively studied and their potentials in remediation were tested: as an example, AgNPs were assayed for remediation of aquatic environments from azo dyes and TiO_2_NPs were explored in converting organic pollutants to environmentally friendly by-products [3, 4]. FeNPs were also considered attractive candidates, since FeNPs can remove or convert various pollutants such as chlorinated organic compounds (e.g., DDT; 1,1,1-thrichloro-2,2-bis(*p*-chlorophenyl) ethene [5]), certain heavy metals (e.g., As and Hg [6]), and toxic inorganic compounds (e.g., nitrate [7]) to inert or non-harmful products. FeNPs exhibit unique size-dependent features such as large specific surface area [8], excellent stability [9], high reactivity [10], outstanding recyclability [11, 12], and relatively easy production [13] and functionalization [14], and the huge NP surface ensures the physical interactions and chemical reactions with the contaminating compounds, where efficient adsorption or degradation of pollutants can occur [15].

Owing to the fact that nanoparticle synthesis is fairly easy and the demand for NPs grows exponentially, these raised some concerns among environment-conscious government legislation and the scientific community. They claim that sustainable production strategies, and greener and ecologically acceptable methods are required for the synthesis of highly efficient nanomaterials, particularly in cases when the material is destined for the treatment and remediation of groundwater and soil [16, 17]. Until recently mainly traditional chemical methods—using harsh reducing and stabilizing chemicals (e.g., sodium borohydride, hydrazine, sodium dithionite, aromatic amines, and thiols)—and some physical techniques were applied to generate nanomaterials [18, 19]. As a matter of fact, the synthesis of FeNPs is most frequently carried out by the reduction of iron(II) or iron(III) salts via sodium borohydride; however, decomposition of iron compounds, vacuum-sputtering, and ball-milling approaches are also quite common [20]. Regarding the impressive latest advancements of nanotechnology, novel eco-friendly alternative approaches with low environmental footprint have gathered grounds, where mild experimental conditions, gentle solvents, environment-safe reducing and capping materials originating from biological entities such as plant extracts, lysates of bacteria or fungi are employed in favor of dangerous chemicals to produce metallic nanoparticles (of iron, but also of silver, zinc, gold, copper and many more), which concomitantly reduces the accumulation of hazardous wastes [21, 22]. This way biopolymers, carbohydrates, polyphenols, and surfactants of the biological entity provide the reduction and stabilization pressure upon nanoparticle synthesis [23, 24]. Particularly, plants are easily accessible for green nanoparticle synthesis since plant-based phytochemicals, e.g., as flavonoids, phenols, sugars, and tannins, would inherently act as either reducers or stabilizers yielding metal NPs with desirable structural properties for a wide range of chemical and biological applications [25, 26].

Based on this knowledge, we have carried out and published a study on an environmentally benign, scalable FeNPs synthesis method where GT, CA, and VC extracts were utilized for FeNPs production [27]. We compared the performance of the obtained green FeNPs with the activity of iron nanoparticles produced by sodium borohydride and sodium dithionite on actual groundwater samples collected from a chlorinated hydrocarbon-contaminated industrial area [28]. Our results revealed that green FeNPs are also capable of degrading chlorinated organic molecules, nevertheless, with somewhat lower efficiency than sodium borohydride and sodium dithionite-derived counterparts. Despite the slightly weaker performance, plant-based green synthetized NPs will definitely play a significant role in future remediation approaches. Even more important would be the utilization of plant wastes instead of fresh plants for NP generation. Given that a large amount of agricultural wastes—plant crops, leaves, or roots left behind agricultural procedures—as well as plant-based household wastes such as coffee grounds in discarded capsules, teabags, vegetable and fruit remains accumulate worldwide every year and the management of this kind of waste is not fully resolved. These could be suitable for the production of metal nanoparticles. The benefits of such approaches—especially from an ecological point of view—are multiple, since not only tons of green household/agriculture wastes can be turned into a product such as FeNPs, but it would enable immediate environmental applications of the nanoparticles produced. In fact, waste management coupled with groundwater and soil remediation via green FeNPs is the ultimate 2-in-1 eco-friendly solution. These approaches highlight the value and the potential of green waste materials and offer further avenues for waste management, and the produced green nanoparticles would, without doubt, be safe and biocompatible alternative agents for wastewater treatment.

On these grounds, the aim of this study was to find out whether the green materials (GT, CA, and VC) extracted and used for the synthesis of nanoparticles could be recycled for further NP synthesis and to reveal if the second- and third-round extracts contain sufficient phytochemicals to reduce and stabilize nanoparticles. Another objective was to examine the structural or functional differences between the FeNPs obtained with the second or third-time recycled green wastes and those FeNPs that were produced with the very first extract. And as a proof-of-concept, we explored the performance of the FeNPs generated with the 1, 2, or 3 times recycled GT, CA, and VC residues in the degradation of VOC to estimate the potential of these green FeNPs for future remediation purposes.

## Materials and methods

All solvents and chemicals were of high purity and were used as received. Most solvents and chemicals were obtained from Merck KGaA (Darmstadt, Germany, Europe); however, if a given chemical was obtained from another company, it is always specified accordingly in the text and the company name is indicated behind the chemical name at its first appearance in the text.

### Preparation of plant extracts in multiple rounds

The various plant extracts were prepared according to previously described procedures [26, 27]. Virginia creeper (*Parthenocissus quinquefolia,* VC) leaves were collected locally (Szeged, Hungary) in April 2020 (mature period), washed thoroughly with deionized water to eliminate surface dust and dried at room temperature until achieving constant weight at ambient conditions. Thereafter, the VC leaves were milled with an electric grinder (Retsch SM 100 Haan, Germany) and sieved to remove the plant particles larger than 2 mm. During first-round extraction, the extracts were prepared by soaking 5 g of dry VC leaves in 100 mL deionized water at 80 °C for 20 min, and then the extracts were paper- and vacuum-filtered (pore size of the membrane was 0.25 µm) to remove residual plant particles. The extracts were stored at 4 °C for further use. A similar process was employed for green tea (GT) and coffee (CA) extracts, except that purchased dry tea leaves (TwiningsTM of London, Green Tea and Lemon) and coffee (Tchibo Family, coffee arabica) were boiled directly without any pretreatment.

After the first-round extractions, residual plant materials (used upon the first extraction, left behind as “waste”) were air-dried and recycled two further times to obtain individual extracts each time (second- and third-round extractions). In these multiple rounds, the extraction method was identical as described above for first-round extractions. The extracts formed were denoted according to the name of the plant extract and the sequence number of the extraction round, namely CA1, CA2, and CA3 for coffee, GT1, GT2, and GT3 for green tea, and VC1, VC2, and VC3 of Virginia creeper extractions, respectively. These extracts were all stored at 4 °C, then characterized and applied separately for iron nanoparticle synthesis.

### Phytochemical analysis

The plant materials were qualitatively examined for the presence of various phytochemical constituents such as flavonoids, phenols, tannins, alkaloids, glycosides, sterols, saponins, and reducing sugars. Phytoconstituents were identified by characteristic color changes and precipitation reactions using standard procedures presented earlier by Mujeeb et al. [29]; details are provided in the Supplementary material (S1).

### Synthesis of FeNPs

GT-Fe-, CA-Fe-, and VC-Fe-labeled green iron nanoparticles were synthesized by adding the corresponding extracts (of the first-, second-, and third-round extractions: GT1, GT2, GT3, CA1, CA2, CA3 and VC1, VC2, VC3, respectively) to 0.10 M aqueous FeCl_3_*6H_2_O solution in a 1:1 volume ratio at room temperature, pH 7 by continuous stirring for 24 h under a nitrogen atmosphere (creating GT1-Fe, GT2-Fe, GT3-Fe, CA1-Fe, and so on) [27]. Thereafter, the prepared nanoparticles were washed with distilled water and ethanol. The freshly prepared colloid samples were used immediately for further experiments.

FeNPs obtained by classic chemical synthesis (SB-Fe) were produced upon reducing the same iron salt by sodium borohydride (NaBH_4_) according to the methods described previously [27]. SB-Fe were applied in parallel experiments in order to compare their characteristics and performance with those of the green FeNPs samples.

### Characterization of the plant extracts and the obtained FeNPs

#### Total phenolic content

The total phenolic content of extracts was determined using the Folin–Ciocalteu method, which is based on the oxidation of phenolic groups with phosphomolybdic and phosphotungstic acids [30]. In each case, the reaction mixture was prepared as follows: 0.020 mL extract solution, 1.58 mL water, and 0.10 mL Folin–Ciocalteu reagent were mixed well. After 4 min, 0.30 mL sodium carbonate solution was added and then the mixture was incubated at 20 °C for 2 h. The absorbance of each sample was determined at 765 nm. Gallic acid solution was used as a reference standard, and the results were expressed as mg gallic acid equivalent/L (mg GAE/L) of the extracted plant material solutions. The total phenolic contents were analyzed in triplicates in all cases.

#### Sugar and citric acid content

Gas chromatography-mass spectrometry (GC–MS) was used to determine the amount of different sugars and citric acid in our green extracts. An Agilent 6890N GC–MS (5975 MSD) (Agilent Technologies, Santa Clara, USA) was applied, and samples were quantified using a GC–MS technique that incorporated a derivatization step designed to convert carboxyl and hydroxyl functional groups to trimethylsilyl esters and ethers, respectively. All samples were diluted 100-fold with HPLC-grade water. Then, 10 µL of diluted samples was placed in a borosilicate glass vial together with 10 µL of internal standard d-glucose-13C-6 solution (70 µg/mL). The solvent was evaporated by CentriVap (Labconco CentriVap Concentrator) at 10 mbar, and 20 µL of freshly prepared methoxyamine hydrochloride solution (20 mg/mL in pyridine) was added to the residue and incubated at 40 °C for 45 min. The sample was then derivatized by adding 50 µL of bis(trimethylsilyl)trifluoroacetamide (BSTFA) (containing 1% trimethylchlorosilane (TMCS) as a catalyst), capping the vial, and heating to and incubating at 70 °C for 90 min. After cooling to room temperature, samples were mixed and analyzed using a GC–MS fitted with a Restek Rxi 5Sil MS column (30 m × 0.25 mm × 0.25 µm). The operating conditions were as follows: the injection port temperature was 280 °C; oven temperature program: the oven was 40 °C for 12 s, ramped at 30 °C/min to 160 °C (hold 2 min) then ramped at 10 °C/min to 280 °C with a 10 °C/min (hold 3.8 min), for a total runtime of 22 min; helium carrier gas flow rate was 1.5 mL/min. MSD was operated in SIM/Scan mode within a 70–400 m/z range. The compounds were identified using the NIST Spectral Search Program v. 2.0f. Calibration curves were constructed by analyzing aliquots of a stock solution of authentic standards that had been evaporated and derivatized in the fashion described above. Quantitation was performed on extracted ion chromatograms. All reported concentrations were corrected for procedural blanks.

#### Determination of the catechin content

High-performance liquid chromatography (HPLC) measurements were performed on the three green tea extracts (GT1, GT2, and GT3) to determine the amount of catechins per sample. HPLC-grade solvents (water, acetonitrile, trifluoroacetic acid) were purchased from VWR International (Radnor, PA, USA). Green Tea Catechin Mix reference substances, including (−)-gallocatechin, (+)-catechin, caffeine, (−)-epicatechin, (−)-gallocatechin-3-gallate, (−)-epigallocatechin-3-gallate, (−)-catechin-3-gallate, and (−)-epicatechin-3-gallate, were obtained from Sigma-Aldrich (Cerilliant, USA). The identification of the peaks on chromatograms was achieved by comparing their retention times and UV spectra (between 200 and 700 nm) with standard levels. Quantitative analysis of identified catechins was performed based on external calibration. The HPLC measurements were performed on a Shimadzu Prominence HPLC system (Shimadzu Corporation, Kyoto, Japan) equipped with a system controller (CBM-20A), a solvent degasser (DGU-20A), a binary pump (LC-20AB), an autosampler (SIL-20AC), a column thermostat (CTO-20AC), and a UV–Vis PDA detector (SPD-M20A) with a 10-mm optical path length flow cell. The acquisition and processing of chromatographic data were performed using LabSolution chromatographic data software (Shimadzu Corporation, Kyoto, Japan). The chromatographic separations were achieved under reversed-phase conditions on a Kinetex C18 (100 mm × 4.6 mm, 2.6 μm, Phenomenex, USA) column. The mobile phase consisted of water (A) and acetonitrile (B) both containing 0.1% (v/v) trifluoroacetic acid. A flow rate of 1.0 ml min^−1^ and a column temperature of 25 °C with 20-µL injection volume were applied with the following gradient program: 1 min, 5% B; 12 min, 40% B; 13 min, 5% B.

#### Characterization of FeNPs

The crystal structure and phase of nanoparticles were verified by X-ray powder diffraction (XRD) using a Rigaku MiniFlex II powder X-ray diffractometer (Rigaku Corporation, Tokyo, Japan) with a Cu Kα irradiation source. The scanning rate was 2°/min over a 2*θ* range of 10°–80°. Fourier-transform infrared spectroscopy (FT-IR) was performed on the biological extracts and on the FeNPs samples obtained by the application of these multiple times extracted green solutions to identify functional groups of chemical components in the samples. A Bruker Vertex 70 spectrophotometer equipped with a Hyperion FT-IR microscope was used to reveal the presence of biofunctional moieties in the plant extracts. All samples were measured by using the reflectance mode of the equipment. The extracts were drop-cast and air-dried on a clean, highly reflective aluminum surface. The FT-IR spectra were collected at a spatial resolution of 4 cm^−1^, between 4000 and 550 cm^−1^, respectively. To assess the morphological characteristic (size and shape) of the green FeNPs, transmission electron microscopic (TEM) images were captured by a FEI Tecnai G2 20×-Twin instrument (FEI Corporate Headquarters, Hillsboro, OR, USA) at an accelerating voltage of 200 kV.

#### Performance and efficiency of FeNPs in degrading chlorinated volatile organic compounds

The degradation potential of the various green iron nanoparticles during reductive dehalogenation of chlorinated volatile organic compounds (VOC) was investigated. Batch tests were conducted to assess trichloroethylene (TCE) degradation efficiency of samples. TCE-spiked (60 mg/L) 50 mM EDTA-containing acetate buffer (50 mM, pH = 5) was reacted with FeNPs samples (2 g/L) in a 20-mL headspace vial under nitrogen atmosphere, and TCE degradation was measured after 4 h incubation at 25 °C. The concentrations of trichloroethene (TCE), *cis*-dichloroethene (cDCE), and vinyl chloride (VCl) were measured using an Agilent Technologies 6850 GC/5975C VL MSD—CTC HS Pal autosampler system equipped with a J&W DB-VRX column (60 m × 0.25 mm × 1.4 μm). Further details of this analysis are provided in our earlier study [28].

## Results and discussion

### Examination of the plant extracts obtained upon each round of extraction

A green synthesis process was applied to produce FeNPs with the help of plant extracts obtained by multiple recycling rounds of green household waste materials. Prior to nanoparticle synthesis, we wanted to explore the possible differences in the composition of our extracts produced in multiple rounds to assess the suitability of these extracts for nanoparticle generation. For this, phytochemical constituents were identified and quantified by numerous techniques, such as FT-IR-based analysis and total phenolic and sugar content measurements, since biochemical components found in plants, such as flavonoids, phenols, sugars, and tannins, were proven to be effective in the bioreduction of metal ions into nanoparticles. A special emphasis was placed on the differences in qualitative and quantitative compositions between the extracts obtained by each subsequent extraction process to reveal the changes in key components potentially influencing FeNPs synthesis and attenuating the efficiency of the obtained FeNPs in the dehalogenation of chlorinated volatile organic compounds.

#### FT-IR-based extract analysis

**Fig. 1:**
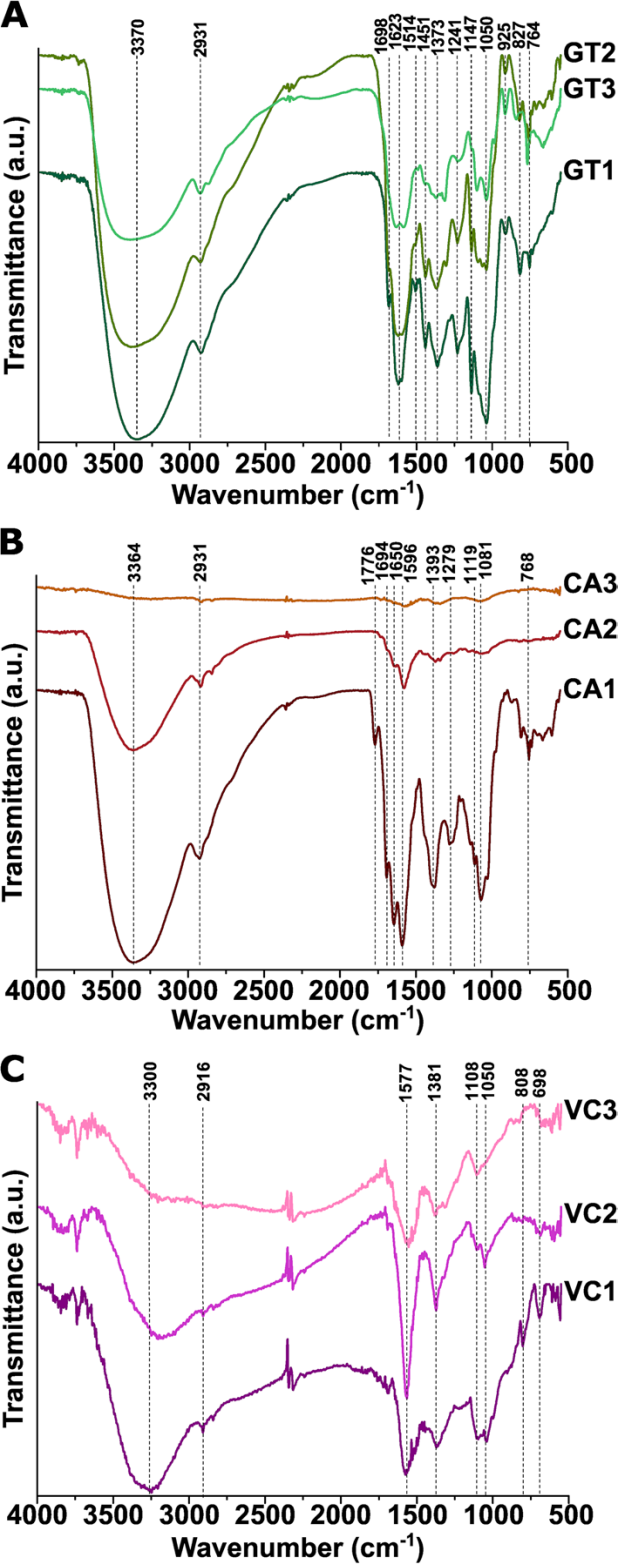
FT-IR spectra of extracts obtained by first, second, and third extraction rounds of green tea (GT1–GT3) (**A**), coffee (CA1–CA3) (**B**), and Virginia creeper extract (VC1–VC3) (**C**)

FT-IR analysis was performed on all the extracts of green tea, coffee, and Virginia creeper produced in multiple rounds to identify the chemical components and functional groups of the samples (Fig. 1). The broad band around 3300–3400 cm^−1^ can be linked to the O–H vibrations of the alcoholic, phenolic, and carboxylic groups in all the samples, as well as to the N–H stretching vibrations in primary and secondary amines and amides. It is visible that the intensity of this peak decreases after the second and third extraction indicating that the number of chemicals containing this group decreases during the repeated extractions. Right next to it, the peak around 2900 cm^−1^ can be linked to the stretching vibration of the C–H groups in the ring of plant sugars and alkanes. The peak between 1570 and 1650 cm^−1^ originates from the C=O stretching vibrations of carbonyl groups indicating the presence of esters, aldehydes, and ketones in the biological samples. In the case of GT and CA samples, multiple, less intense peaks are visible around 1600–1700 cm^−1^. Surprisingly, in the case of VC samples, instead of the multiple peaks observed in this region by the other two samples, only one peak can be identified. It is possibly caused by a small variety of these molecules in the VC plant compared to the others. The peak around 1370–90 cm^−1^ is attributed to C–H bending vibrations and the broad, high-intensity peak between 1050 and 80 cm^−1^ can be linked to the C–O–C stretching vibrations of polysaccharides and aromatic ethers found in the plants and extracts [26, 31]. The multiple peak region between 920 and 700 cm^−1^ is attributed to the –NH_2_ wagging vibration of N-containing molecules, such as amines, as well as different C–H out-of-plane vibrations of substituted organic compounds.

Reduced intensity of these characteristic vibration bands following repeated extraction can be assigned to the reduced amount of water-soluble compounds left in the dried plant material. We found the smallest decrease in intensity in the band around 3300–3400 cm^−1^ and around 1600–1700 cm^−1^, indicating the presence of –OH and –C=O groups possibly from polyphenols, carboxyl acids, ketones, aldehydes, or alcohols after the multiple extractions. By comparing the FT-IR pattern changes upon subsequent extraction rounds of the three plant materials, we observed that while in the case of GT and VC extracts a similar trend in pattern change was detected, CA extracts and their FT-IR spectra changed differently. Namely the peaks characteristic for the components present after the first extraction of GT and VC can be observed in the extracts obtained in the second and third rounds, even with reduced intensity (Fig. 1A, C). These results imply that these green household, agricultural and industrial waste materials can be effectively utilized multiple times and the obtained extracts using the subsequent extraction steps contain sufficient levels of the key components to possibly generate FeNPs. However, for coffee extracts significantly lower intensity peaks were observed already following the second extraction round, which indicates somewhat reduced amounts of water-soluble compounds that would presumably be lacking upon nanoparticle synthesis (Fig. 1B).

#### Phytochemical analysis

The rapid phytochemical testing revealed that all of the aqueous extracts containing a number of phytochemicals, i.e., the presence of flavonoids, phenols, terpenoids, and saponins, were verified. Glycosides were present only in green tea and coffee extracts (Table S1). The role of these above-mentioned constituents in nanoparticle synthesis has already been confirmed [32]. The assays also showed that alkaloids and tannins were absent from all the samples. The lack of these molecules was surprising; nevertheless, it cannot be completely excluded that some small amount of alkaloids and tannins were actually present in the green extracts that were subsequently prepared for nanoparticle synthesis, since in that case the long boiling step could enhance the extraction of these compounds.

#### Total phenolics and sugar content analysis

As described in the literature, various phenolic components, reducing sugars, and citric acid can play an important role in nanoparticle synthesis, since these frequently act as reducing and stabilizing agents alone or combined with other reducing agents during these processes [32, 33]. Therefore, total phenolics, main sugar contents, and the amount of citric acid were determined for each plant extract obtained from green tea, coffee, and Virginia creeper in all three aqueous extraction rounds.

Based on the total phenolic content measurements, the GT samples showed the highest concentrations of total phenolics in each extraction round compared with CA and VC extracts (in fact multiple times higher than in CA and VC samples) (Table 1). The GT1 sample showed the highest total phenolic content (5332.67 ± 183.3 mg GAE/L), followed by CA1 (3346.66 ± 15.28 mg GAE/L) and VC1 (1004.61 ± 7.57 mg GAE/L) samples. Considering these results, the extract with the highest phenolic content will presumably be the most desirable for FeNPs generation. These findings were corroborated with a report by Cyboran et al. on characterizing the phenolic compounds of these plants [34]. For each biological source, the total phenolics content decreased with repeated extraction. Further, the total phenolics content of green tea samples decreased significantly in the second (2603.45 ± 20.82 mg GAE/L) and third rounds (1306.21 ± 45.83 mg GAE/L); however, their levels were notably higher than CA and VC samples in the first round. These data imply that GT samples contain large amounts of phenolic components even after multiple extractions. In coffee samples, the total phenolic content was reduced to 737.27 ± 3.46 mg GAE/L in the second and to 345.30 ± 7.81 mg GAE/L in the third extraction rounds. These values are comparable in magnitude to those of VC samples (VC2: 465.97 ± 15.37 mg GAE/L; VC3: 212.54 ± 5.29 mg GAE/L). VC samples showed the lowest amounts of total phenolics in the solutions obtained by each round of extraction.

**Table 1:** Total phenolics, sugar, and citric acid content of extracts obtained by first, second, and third extraction rounds of green tea (GT1–GT3), coffee (CA1–CA3), and Virginia creeper (VC1–VC3)

Waste extract	Round#	Total phenolics (mg GAE/L)	Fructose (mg/L)	Glucose (mg/L)	Sucrose (mg/L)	Mannitol (mg/L)	Citric acid (mg/L)
Green tea	1	5332.7 ± 183.3	82.2 ± 1.2	137.0 ± 1.8	532.0 ± 2.6	2.1 ± 0.1	50.8 ± 1.0
	2	2603.5 ± 20.8	15.4 ± 0.3	25.2 ± 0.7	75.5 ± 1.1	n.d	8.4 ± 0.1
	3	1306.2 ± 45.8	2.5 ± 0.2	6.2 ± 0.1	20.6 ± 0.6	n.d	< 1.5
Coffee arabica	1	3346.7 ± 15.2	n.d	n.d	2.4 ± 0.1	2.8 ± 0.1	108.0 ± 0.8
	2	737.3 ± 3.4	n.d	n.d	< 1.5	< 1.5	5.2 ± 0.1
	3	345.3 ± 7.8	n.d	n.d	< 1.5	< 1.5	< 1.5
Virginia creeper	1	1004.6 ± 7.5	203.0 ± 1.1	214.0 ± 1.0	2.1 ± 0.1	7.8 ± 0.1	31.4 ± 0.2
	2	466.0 ± 15.3	19.3 ± 0.1	17.1 ± 0.2	n.d	n.d	3.5 ± 0.1
	3	212.5 ± 5.2	< 1.5	1.7 ± 0.1	n.d	n.d	< 1.5

Detection limit: 1.5 mg/L

Regarding the sugar content analysis, the extracts obtained from the three biological entities revealed a large difference also in the distribution as well as the amount of certain carbohydrates. While GT samples contained all tested sugar types in detectable quantities, VC samples were comprised mainly of just glucose and fructose. Interestingly, only mannitol and sucrose could be detected in CA samples, and not surprisingly the quantity of these water-soluble carbohydrates gradually decreased with repeated extractions. After the second round of extraction, the obtained CA sample did not show any sugar content. In the case of GT and VC samples, carbohydrate quantities originally present in the first-round samples dramatically decreased after repeated extraction of the remnant biological material. Nevertheless, for GT, the sugar amount of the obtained extracts was still significant, even after the third extraction round. This latter finding might be relevant considering the FeNPs synthesis, where various carbohydrates can act as reducing and stabilizing agents.

To overcome the obstacle of particle agglomeration, and thus a decrease in the surface area/volume ratio leading to a reduction in FeNPs effectiveness, organic compounds, such as citric acid or chitosan, have been frequently employed for preparing nanoparticles [35]. These compounds coat the nanoparticles, thereby stabilize them and prevent their oxidation in aqueous media. Since citric acid can be present in its natural form in various plants, mainly in fruits, we examined the citric acid content of the extracts. Based on the analysis, the amount of citric acid originally present in first-round samples gradually decreased after repeated extractions of the remaining biological material. Citric acid was still detectable in second-round extracts, albeit it was basically lacking from the third-round samples indicating that no extractable citrate remained within the waste materials.

These values correspond to the data presented by Mizukami et al. and Ding et al. published on the various carbohydrate concentrations of several types of tea leaves; nevertheless, it has to be noted that the sugar content of the green source materials might vary greatly between the region of cultivation and depend on the weather and climate specifics of the area [36, 37].

#### Analysis of catechins

According to the literature data, certain catechins (a subgroup of polyphenols called flavonoids) are present in the majority of green extracts and play a definitive role in particle synthesis [32, 38]. Based on the results obtained in FeNPs synthesis (described in detail in “Examination of the suitability of the plant extracts obtained in multiple recycling rounds to form iron nanoparticles” section), clearly, green tea extracts (GT1-3) proved to be the most suitable for particle production. Therefore, we performed further examination to determine the amounts of these components in our extracts. An HPLC method was used to determine the catechin composition of green tea extracts (Table 2), where a calibration series of green tea catechin standards was established and the catechin concentrations of the samples were then measured. Retention times of phenolic compounds, which have been previously described for the tea extract (Table 2), were determined using the aforementioned elution program as part of a multicomponent analysis [39]. Chromatograms corresponding to the tea extract showed eight intensity peaks at 3.59, 5.36, 5.45, 6.13, 6.40, 6.71, 7.52, and 7.68 min. Although it is known that the water solubility of polyphenolic compounds is low, the presence of seven different catechins ((−)-gallocatechin, (+)-catechin, caffeine, (−)-epicatechin, (−)-gallocatechin-3-gallate, (−)-epigallocatechin-3-gallate, (−)-catechin-3-gallate, and (−)-epicatechin-3-gallate) were verified in all green tea extracts. It is also evident that the concentrations of these flavonoids decreased upon subsequent extractions. Moreover, most gallo- and epicatechins were extracted in the first two extraction rounds, while in the third-round extract only caffeine can be detected in a larger quantity. These results are in good agreement with the current literature data and are in line with the previously shown FT-IR and total phenolics content values for these samples, where the subsequent extraction steps resulted in a downward trend in the amount of phenolics components [39].

**Table 2:** Catechin profile of green tea samples obtained at three subsequent extraction rounds

Peak number	Component	Retention time (min)	Concentration (μg/mL)		
			GT1	GT2	GT3
1	(−)-gallocatechin	3.59	221.9	44.3	4.8
2	(+)-catechin	5.36	44.9	16.9	1.3
3	caffeine	5.45	773.1	228.8	88.9
4	(−)-epicatechin	6.13	413.5	86.3	5.6
5	(−)-gallocatechin-3-gallate	6.40	1400.3	234.2	9.2
6	(−)-epigallocatechin-3-gallate	6.71	106.4	43.6	6.0
7	(−)-catechin-3-gallate	7.52	352.8	120.4	7.3
8	(−)-epicatechin-3-gallate	7.68	–	–	–

### Examination of the suitability of the plant extracts obtained in multiple recycling rounds to form iron nanoparticles

Following the procedure described earlier, we performed iron nanoparticle synthesis with the help of each extract obtained in the multiple rounds of extraction [27]. Our main aim was to examine the suitability and reusability of green household waste materials for FeNPs generation and to identify which green material yields sufficient quantity and quality of key components after successive extraction steps to provide FeNPs. Upon nanoparticle synthesis in some cases, we observed a color change when certain aqueous green extracts were added to the reaction mixture. This color change varied in tint and manner; the conversion was from yellowish/greenish to green–brown, brown, or black. The latter one indicated that the initial iron salt was being bioreduced; thus, the iron ions in the solution were reduced to form FeNPs [40]. From these color changes, we assumed that not all nanoparticle synthesis reactions were successful. XRD and TEM measurements were carried out as a primary characterization procedure, since crystal structure, particle shape, and size distribution are key features to verify the success or the failure of the nanoparticle syntheses. Each feature of the generated green FeNPs was compared to that of SB-Fe nanoparticles prepared by conventional chemical reduction using sodium borohydride.

#### XRD measurements

**Fig. 2:**
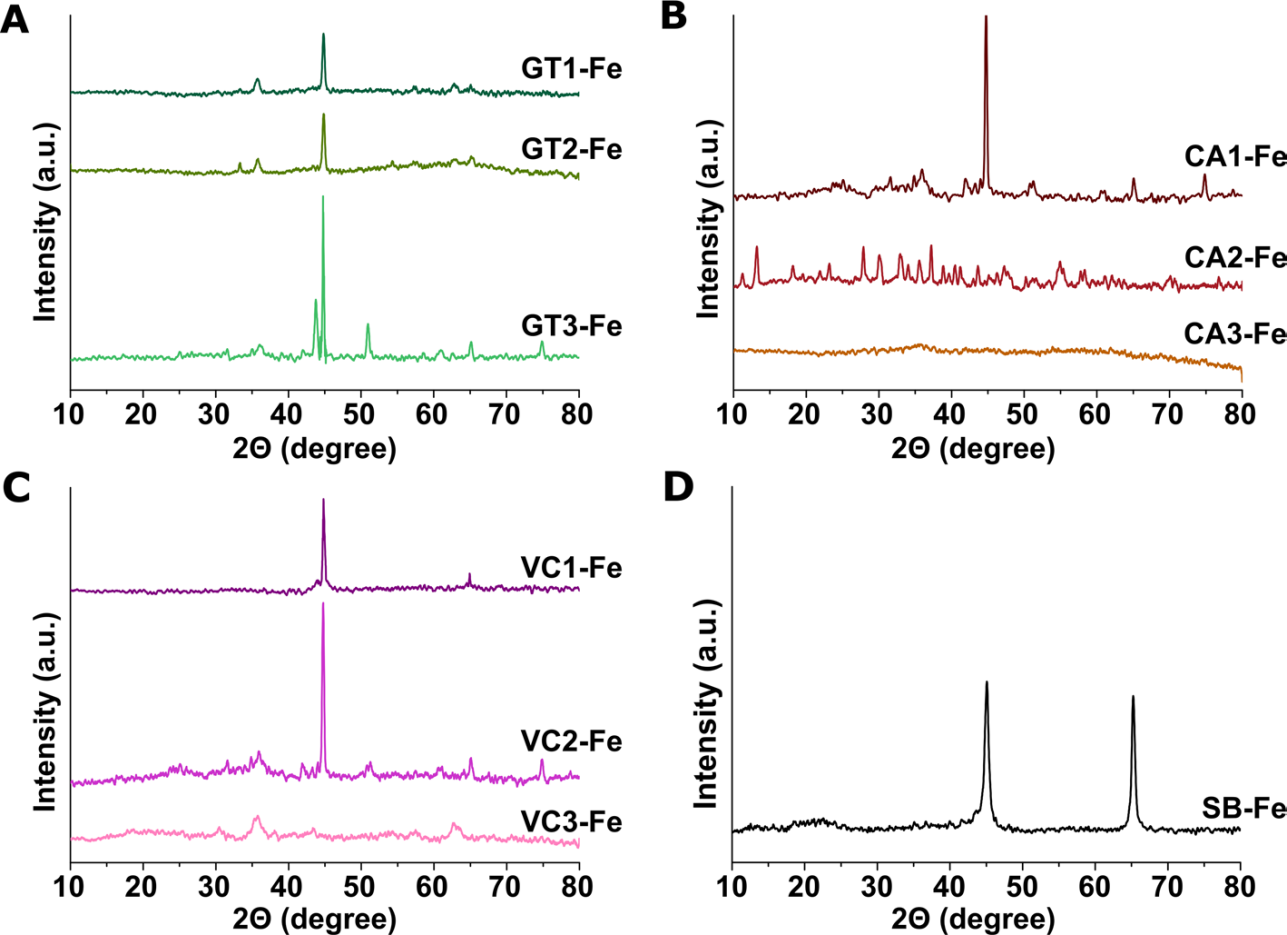
X-ray diffraction patterns of iron nanoparticles produced by using various green waste extracts obtained in multiple extraction rounds. Iron nanoparticles were synthesized by three recycling extraction rounds of green tea extracts (GT1-Fe, GT2-Fe, and GT3-Fe) (**A**), coffee (CA1-Fe, CA2-Fe, and CA3-Fe) (**B**), Virginia creeper (VC1-Fe, VC2-Fe, and VC3-Fe) (**C**), and by chemical method using sodium borohydride (SB-Fe) (**D**)

XRD studies were performed to confirm the material structure of the synthesized iron nanoparticles. Figure 2 shows the XRD patterns of freshly synthesized FeNPs. The reflections at 2Θ of 45° and 65° can be assigned to the (110) and (200) planes of metallic iron [27, 41]. No iron oxide peaks were measured for the freshly prepared samples of GT1-Fe, VC1-Fe, and SB-Fe. Other minor reflections in diffractograms of the other samples signify the iron oxide and iron hydroxide phases. These latter XRD features are most pronounced for the second- and third-round extracts, especially for CA2-Fe and VC3-Fe samples.

Based on these XRD analyses we found that not all extracts were suitable for FeNPs generation, whereby nanoparticles were obtained only when GT1, GT2, GT3, CA1, VC1, and VC2 extracts were applied (therefore only these data are shown hereafter, and those belonging to SB-Fe). These results were in accordance with the color changes observed during nanoparticle synthesis events and also with the lack of color change in certain cases.

#### FT-IR analysis of FeNPs

**Fig. 3:**
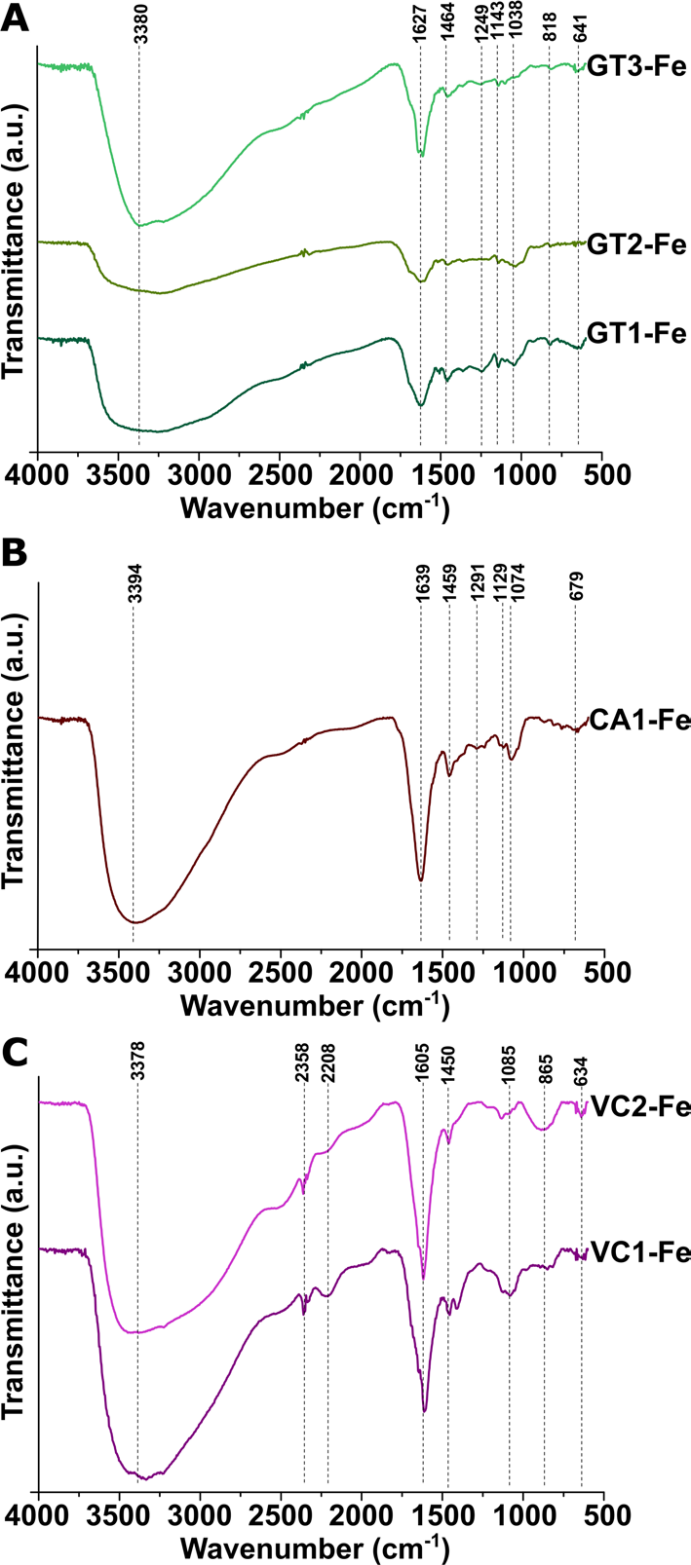
FT-IR spectra of iron nanoparticles produced by using various green waste extracts. Spectra of FeNPs generated with green tea solutions of three subsequent extraction rounds (GT1-Fe, GT2-Fe, and GT3-Fe) are shown in panel **A**, the spectrum of FeNPs obtained with first-round coffee extract (CA1-Fe) is presented in panel **B**, and FT-IR spectra of FeNPs of first and second extractions of Virginia creeper (VC1-Fe, VC2-Fe) are shown in part **C**

To elucidate the functional groups present on the surface of the nanoparticles obtained by using green waste extracts from multiple extraction rounds, the FT-IR spectra of FeNPs were investigated. As is observable in Fig. 3, each nanoparticle sample yielded relatively similar spectra (spectra obtained for GT1-Fe, GT2-Fe, GT-Fe are shown in Fig. 3A, for CA1-Fe in Fig. 3B, and for VC1-Fe and VC2-Fe in Fig. 3C respectively). In each case, peaks can be seen between wavenumber 3400 and 3430 cm^−1^, which correspond to the O–H stretching of phenolic groups [26]. These peaks show a blueshift compared to the peaks of the FT-IR spectra obtained on the corresponding plant extracts. This result was expected since it is generally believed—and as was mentioned also in “FT-IR-based extract analysis” section—that these phenolic compounds are responsible for chelation, capping, and reduction of the ferric ions [31]. On the other hand, somewhat surprisingly, the peak around 2900 cm^−1^, which can be attributed to the stretching vibration of the C–H groups in the ring of plant sugars and alkanes, disappeared from the spectra of each nanoparticle. The peaks at 1627, 1639, and 1605 cm^−1^ in GT-, CA-, and VC-mediated samples, respectively, are due to the C=O stretching vibrations of carbonyl groups. The peak between 1450 and 1465 cm^−1^ originates from the C–C and C=C aromatic stretching; moreover, those between 1040 and 1085 cm^−1^ can be linked to the C–O–C stretching vibrations of polysaccharides and aromatic ethers [26, 31]. These bands were also found in the plant extracts, although there was a slight shift in the observed vibrational frequencies. The peaks at 641 cm^−1^, 679 cm^−1^, and 634 cm^−1^ in GT-, CA-, and VC-FeNPs are assigned to Fe–O vibrations [42].

These findings are in agreement with the results of previous studies, where similar observations were made. Comparisons between the FT-IR spectra of FeNPs and the corresponding green extracts were in correlation and indicated that the phytochemicals of the plant extracts contributed to the synthesis and capping of FeNPs. In addition, Wanakai et al. reported that the shift of bands observed between the plant extracts and FeNPs, the reduced intensity, and the disappearance of some characteristic bands indicate that water-soluble biomolecules such as polyphenols and reducing sugars are responsible for the biological reduction of ferric ions [42]. These data were consistent with our findings and further strengthened our results obtained by the phenolic and sugar content measurements.

#### TEM measurements

**Fig. 4:**
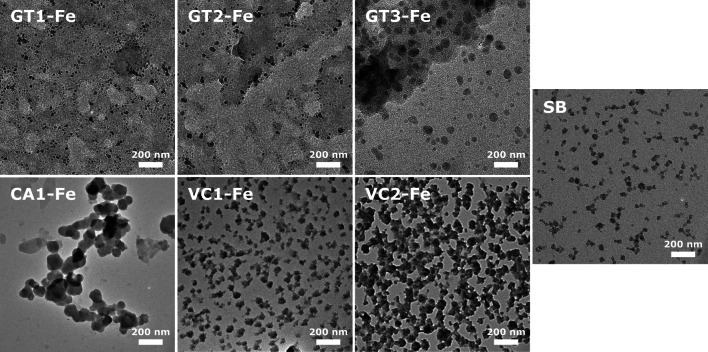
TEM images of iron nanoparticles produced by using various green waste extracts obtained in three extraction rounds of green tea (GT1-Fe, GT2-Fe, and GT3-Fe), first round of coffee (CA1-Fe), first and second rounds of Virginia creeper (VC1-Fe, VC2-Fe), and by chemical method using sodium borohydride (SB-Fe)

In addition to the particle crystal structure, shape and size are important features determining the physicochemical properties of nanoparticles. Therefore, TEM measurements were employed to ascertain the size and morphological characteristics of the obtained FeNPs samples. According to these images (Fig. 4), in each of the six samples where nanoparticle synthesis was successful, FeNPs exhibited isotropic morphology. When the first-round extracts GT1 and VC1 were used, the formed particles were well separated from each other, however, were trapped in the matrix of the residual green waste extract (Fig. 4). In contrast, CA1-Fe particles were larger, minor polydispersity was observed, and the particles formed large aggregates as the organic compounds of the extract did not surround the particles. Nanoparticles obtained with the extracts of the second extraction round, i.e., with GT2 and VC2, were larger compared to the first-round ones and agglomeration was also observed (Fig. 4 upper second and lower third image). FeNPs obtained by GT3 extracts were similar in appearance to CA1-Fe, comprising mainly large agglomerated particles. Based on image analysis, the average particle diameter was around 20.8 ± 5.4, 31.2 ± 11.2, 60.6 ± 13.5 nm for GT1-Fe, GT2-Fe and GT3-Fe, 102.2 ± 33.1 nm for CA1-Fe and 43.1 ± 5.9 and 52.6 ± 10.8 nm for VC1-Fe and VC2-Fe particles, and 29.9 ± 6.1 nm for SB-Fe, respectively. We observed that the nanoparticle size and agglomeration tendency increased with the number of extractions.

A possible explanation for the success of certain syntheses and failure of CA2-, CA3-, and VC3 extract-mediated procedures may originate from the composition of the various extracts. As previously presented, numerous biomolecules are involved in the mechanism of nanoparticle formation using plant extracts, thus many compounds are possibly responsible for the reduction of metal ions [43]. Specifically, a combination of several plant components, such as enzymes, amino acids, proteins, vitamins, polysaccharides, organic acids, polyphenols, and flavonoids, participates in the generation of particles in metal ion-supplemented media. These are biodegradable non-toxic substances that can act both as reducing and capping agents, thus promoting the formation of nanoparticles and inhibiting their agglomeration [32]. Phenolic chemicals and reducing sugars derived from natural sources play also a prominent role in the synthesis of nanoparticles due to their protonating and absorbing capabilities, as well as their antioxidant effects [44]. Their high oxidation ability can promote the initiation of the nucleation process, the reduction of metal salts into metal nanoparticles, and by physical attachment to the surface of the particles they enhance the stability of the obtained colloid.

Although the first coffee extract (CA1) contained polyphenols, their amount decreased dramatically upon subsequent extraction rounds. Moreover, compared with the GT2 and VC2 extracts the quantity and quality of sugars in the CA2 extract were reduced massively, leading to a lower efficiency or no nanoparticle formation. Interestingly, however, the composition of the GT and VC extracts was nearly similar based on the FT-IR spectra. Of all the third-round extracts, only GT3 was capable to yield FeNPs, suggesting that sufficient sugars, phenolics, and catechins were in the extract to reduce and stabilize the obtained nanoparticles.

#### Performance of iron nanoparticles generated by green wastes in the degradation of chlorinated volatile organic compounds

**Fig. 5:**
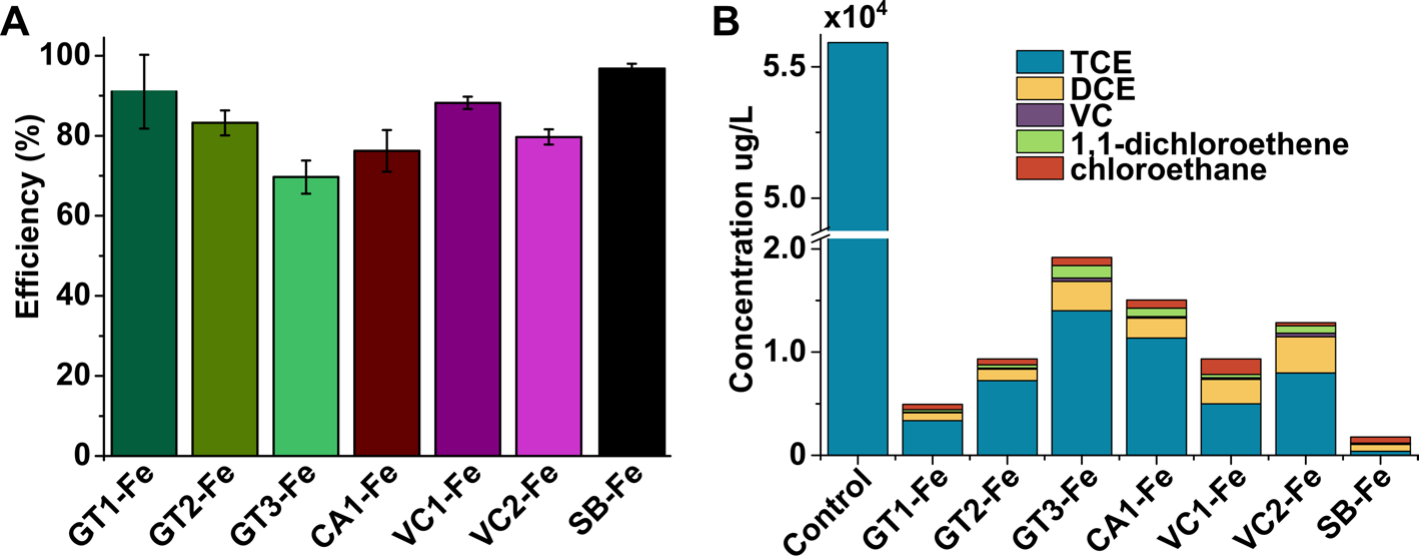
Performance of iron (Fe) nanoparticles produced by using various green waste extracts obtained in multiple rounds and conventional chemical synthesis method. Iron nanoparticles were synthesized using extracts obtained by three extraction rounds of green tea (GT1-Fe, GT2-Fe, GT3-Fe), first round of coffee (CA1-Fe), first and second rounds of Virginia creeper (VC1-Fe, VC2-Fe), and by chemical method using sodium borohydride (SB-Fe). Efficiency of different FeNPs in reductive dehalogenation of VOC (**A**), and concentrations of remaining TCE and its metabolites after addition of different FeNPs (**B**) are depicted in Fig. 5

As we have seen that several green materials can be reextracted in multiple rounds, the biological waste materials can be recycled and the obtained solutions contained sufficient biomolecules to be suitable for FeNPs production, we aimed to characterize the performance of these green iron nanoparticles in the degradation of TCE. From an environment-relevant point of view, it is also essential to know how efficient these green FeNPs are in the reductive dehalogenation of chlorinated VOCs. In this regard, we conducted batch tests to assess the TCE degradation efficiency of the generated nanoparticles (Fig. 5). VOC in an aqueous solution with an initial concentration of 60 mg/L was reacted with FeNPs samples applied in 2 g/L concentration, and the reaction time lasted 4 h. Degradation efficiency was expressed in percentage obtained from the ratio of summed VOC concentrations after 4 h to the initial VOC concentration. An efficiency of 100% would be equivalent to the complete removal of VOCs from the aqueous solution.

Based on our results, the efficiency of VOC degradation was the following: GT1-Fe 91.0%, GT2-Fe 83.2%, GT3-Fe 68.5%; CA1-Fe 76.2%; VC1-Fe 88.2%, VC2-Fe 79.7%, and SB-Fe 97.3% respectively. These results indicate that VOC degradation performance was significantly affected by nanoparticle structure (XRD data) and thus by the utilized synthesis method. Nevertheless, VOC could be eliminated each time a green waste-synthesized FeNPs was employed. As expected, FeNPs prepared by sodium borohydride (SB-Fe) worked slightly better than the green-produced counterparts [27]. Moreover, the FeNPs samples synthesized using first-round green extracts of green tea or Virginia creeper (GT1-Fe and VC1-Fe) exhibited the greatest VOC removal efficiencies compared with other FeNPs. Among the first-round extract-produced FeNPs, the coffee-derived sample (CA1-Fe) manifested the lowest efficiency; however, this was in agreement with previous literature reports [45]. The highest degradation efficiency among green-produced FeNPs was obtained using GT1-Fe, in fact, these particles were comparably effective to the borohydride-mediated chemical control (SB-Fe). According to the analysis of VOC composition (TCE and its metabolites) of the model reaction system, the VOC reduction with green synthetized FeNPs—mainly from the second and third rounds—was incomplete, and some metabolites and intermediates of by-reactions (DCE, VCl, and chloroethanes) remained which have similar toxicity as TCE. Despite the fact that a precise mechanism cannot be identified with certainty, a possible degradation pathway would be the following: TCE reacts to produce c-DCE, t-DCE, and 1,1-DCE simultaneously and these daughter species further react to produce VCl. Monochloroethane usually is a daughter product of 1,1-dichloroethane during reduction. Although the distribution of TCE and its metabolites varied slightly when FeNPs of different plant-derived extracts were applied, the proportion of the various metabolites changed in the same manner when FeNPs prepared with different extracts of the same green material (i.e., GT1-3) reacted with the starting VOC.

Our observations can be explained by the presence of a large number of polyphenols and reducing sugars in all first-round extracts, supported by FT-IR spectra and various composition analyses. These biomolecules are responsible not only for the reduction of iron ions but for the stabilization of the produced nanoparticles as well, consequently, the stability of the nanoparticles generated by GT1, CA1, or VC1 was the highest among all. This was supported by XRD data showing only the peaks related to elemental iron in the case of these nanoparticles (GT1-Fe or VC1-Fe). These findings are also consistent with another previously published study where the degradation of pollutants by green-synthesized FeNPs indicated high lifetime and oxidation resistance of nanoparticles [44, 45]. The green matrix, in which the particles are embedded, can contribute to the particle regeneration process after reacting with pollutants, which may enhance the performance of these eco-friendly nanoparticles, potentially in the long term, and provides additional benefits for their utilization. Even more important are the findings related to FeNPs obtained with the second-round green extracts, since the green materials utilized upon such syntheses are de facto wastes of the first extraction process and their potential recycling for subsequent nanoparticle generations is a key achievement. Remarkably, the performance of FeNPs obtained with GT2 and VC2 extracts in VOC degradation was around 80%. This is more than acceptable considering that these green extracts contained definitely less amount of polyphenols, sugars, and flavonoids than the extracts of the first-round extraction; nevertheless, proper nanoparticle synthesis and adequate VOC degradation potential could be obtained in these cases as well. Thus, such green approaches indeed provide a safer and economical alternative to conventional chemical methods and a low environmental footprint not simply because green materials are utilized for FeNPs production but also because these household and agricultural wastes can be reused over and over again for iron nanoparticle synthesis. What is more, the resulting nanoparticles could be efficiently applied for the degradation of water and soil pollutants, such as VOCs.

To explore the limitations of recycling the green waste materials, we tried FeNPs synthesis with the extracts obtained after three rounds of extractions. Only GT3 was suitable for Fe nanoparticle synthesis. Although the citric acid and sugar content of this extract was significantly lower than GT1 and GT2, the successful FeNPs synthesis via GT3 was probably due to the relatively high polyphenol content of this extract. In fact, the polyphenol amount of GT3 (after three rounds of extractions) was higher than those of VC1 or CA1 (after only one extraction step). As expected, the components of the GT3 extract acted as reducing and capping agents during nanoparticle synthesis; however, the formed particles were larger in size, moreover, exhibited the worst oxidation tolerance, and showed greater aggregation propensity than the other FeNPs of the study, according to TEM images. The VOC degradation efficiency of these GT3-Fe particles was significantly less compared to the other nanoparticles; nevertheless, they exhibited comparable activity to CA1-Fe. The performance of the latter was proclaimed acceptable for remediation, based on a previous publication [46].

Based on these results it is evident that some green materials or their corresponding wastes (e.g., GT) are suitable for multi-round extractions and the green extracts are adequate for multi-round FeNPs production owing to the bioactive components present upon nanoparticle synthesis. This multiple extraction is a feasible way for a more economical utilization of higher polyphenols, sugars, or citric acid-containing plants in the nanoparticle synthesis. Importantly, the obtained FeNPs are highly capable of degrading chlorinated pollutants. This way waste management can be potentially coupled with groundwater and soil remediation via green FeNPs, augmenting the ecological and economic advantages of this strategy. On the other hand, some green materials (CA) are not recyclable, since these yield efficient FeNPs only upon utilizing the first possible green extract. Therefore, based on the proper assessment of environmental and economic aspects and the characteristics of the given contaminated area, a certain green FeNPs sample should be considered for the remediation process, and depending on the circumstances (multiple injections, long-term injection of nanoparticles, combinational injection of nanoparticles with other remediation agents) the most suitable material—maybe even a GT3-Fe—should be employed. Based on the results, we suggest that GT and VC are useful raw materials with plant polyphenols, sugars, and acids that can be extracted even upon multiple rounds of aqueous extraction. With the help of these molecules, iron nanoparticles can be successfully synthesized and the produced particles can be used for reductive remediation purposes.

## Conclusion

Nowadays, nanoparticles are used extensively in various fields of industry and technology such as medicine, solar energy, drug delivery, water treatment, detection and reduction of persistent pollutants. Therefore, there is an urgent need to adopt cost-effective and green technologies for the synthesis of NPs. One possible green solution might involve the utilization of green wastes. Huge amounts of household/agricultural green wastes are generated every year, around 30% of which is recycled or composted. However, a higher rate of recycling this type of waste could be achieved by utilizing secondary waste resources. Numerous independent studies verified that plants are easily accessible and suitable for green nanoparticle synthesis, since plant-based phytochemicals such as polyphenols, flavonoids, sugars, and tannins ensure the reduction and stabilization of the nanoparticles upon synthesis. In this regard, we investigated the multiple applicability, i.e., recyclability of green tea, coffee, and Virginia creeper waste extracts for green FeNPs synthesis. To identify the compounds responsible for particle synthesis, the obtained extracts were characterized by various chemical analyses and then applied successfully for nanoparticle production. All green extracts obtained in the first extraction round were adequate for FeNPs synthesis, but VC and GT plant residues could be utilized multiple times. These green waste materials could be extracted 2 or 3 times, respectively, then yielding iron nanoparticles with appropriate structural and functional features. All these multiple-round achieved GT and VC extract-mediated particles were able to degrade volatile organic compounds and thus are suitable for remediation purposes. These results indicate that the well-chosen green waste material can be reused in multiple rounds for nanoparticle synthesis, serving as a straightforward alternative to chemical methods, and providing economic and social benefits in waste minimization and resource recycling.

## Supplementary Information

Below is the link to the electronic supplementary material.

Supplementary file1 (DOCX 15 KB)

## Data Availability

All original data of this manuscript are available upon request.
