# Supplementary material for: Multi-round recycling of green waste for the production of iron nanoparticles: synthesis, characterization, and prospects in remediation
Source: Discov Nano. 2023 Feb 9;18(1):8. doi: 10.1186/s11671-023-03784-x (PMC9911567; doi:10.1186/s11671-023-03784-x)
Supplement: Supplementary file 1 — Supplementary file1 (DOCX 15 KB) [file 11671_2023_3784_MOESM1_ESM.docx]

**Phytochemical analysis**

The plant materials were qualitatively examined for the presence of various phytochemical constituents such as flavonoids, phenols, tannins, alkaloids, glycosides, sterols, saponins, and reducing sugars. Phytoconstituents were identified by characteristic color changes and precipitation reactions using standard procedures presented earlier by Mujeeb *et al* (Mujeeb F, Bajpai P, Pathak N. Phytochemical evaluation, antimicrobial activity, and determination of bioactive components from leaves of aegle marmelos. Biomed Res Int. 2014; 497606.).

In details:

*Alkaloids.* 200 mg plant material was boiled in 10 mL methanol and filtered. 1% hydrochloric was added followed by 6 drops of Dragendorff reagent, and brownish-red precipitate indicated the presence of alkaloids.

*Flavonoids.* To 2 mL filtrate of plant material 5 mL of dilute ammonia solution was added, followed by concentrated sulfuric acid. A yellow coloration indicated the presence of flavonoids.

*Glycosides.* 2 mL filtrate was treated with 1 mL glacial acetic acid containing few drops of ferric chloride. Concentrated sulfuric acid was added to the mixture. Green-blue color indicated the presence of cardiac glycosides.

*Saponins.* 5 mL distilled water was added to 200 mg plant material. 0.5 mL filtrate was diluted to 5 mL with distilled water and shaken vigorously for 2 minutes. Formation of stable foam indicated the presence of saponins.

*Tannins.*200 mg of plant material was boiled in 10 mL distilled water and few drops of ferric chloride were added to the filtrate; a blue-black precipitate indicated the presence of tannins.

*Terpenoids*. To 200 mg plant material 2 mL of chloroform and 3 mL of concentrated sulfuric acid were added. A reddish-brown coloration indicated the presence of terpenoids.

*Steroids.* 200 mg plant material was added in 10 mL chloroform. Acetic anhydride was added in the ratio of 1 : 1. The formation of blue-green ring indicated the presence of steroids.

*Reducing Sugars.*To the 10 mL of aqueous extract a few drops of Fehling’s solution A and B were added; an orange red precipitate indicated the presence of reducing sugars.

**Supplementary Table 1.** Results of the phytochemical analysis

| **Phytochemicals** | **Green tea** | **Coffee arabica** | **Virginia creeper** |
| --- | --- | --- | --- |
| Alkaloids | - | - | - |
| Flavonoids | + | + | + |
| Glycosides | + | + | - |
| Phenols | + | + | + |
| Saponins | + | + | + |
| Tannins | - | - | - |
| Terpenoids | + | + | + |
| Steroids | - | - | - |
| Reducing sugar | + | + | + |

‘+’: presence; ‘-’: absence
